# Exploring the Difference in Post-procedural Stroke Rates Between Patients with Aortic Stenosis Who Undergo Transcatheter Aortic Valve Replacement Versus Surgical Aortic Valve Replacement

**DOI:** 10.7759/cureus.2494

**Published:** 2018-04-17

**Authors:** Heather Burke, Agnieszka Boron, Jung-Hyun Lee, Kudrit Riana Kahlon

**Affiliations:** 1 Internal Medicine, University of Central Florida College of Medicine, Orlando, USA

**Keywords:** savr, tavr, aortic stenosis, stroke outcomes, aortic valve replacement, transcatheter aortic valve replacement, surgical aortic valve replacement

## Abstract

Transcatheter aortic valve replacement (TAVR) is a newer alternative to surgical aortic valve replacement (SAVR) for patients with severe aortic stenosis. Clinical trials have investigated TAVR's safety and effectiveness. Cerebral embolization is a common complication leading to stroke after TAVR and SAVR; different cerebral protection methods have been studied to prevent this.

This paper evaluates the rate of post-procedural stroke rates between TAVR and SAVR and investigates the effect of cerebral protective methods on the stroke risk post-TAVR.

Publications on TAVR and SAVR were found using specific criteria on PubMed. The Placement of Aortic Transcatheter Valves 1 (PARTNER 1) and PARTNER 2, the Nordic Aortic Valve Intervention (NOTION), and Surgical Replacement and Transcatheter Aortic Valve Implantation (SURTAVI) trials compared the incidence of complications between TAVR and SAVR. The Action in Diabetes and Vascular Disease: Preterax and Diamicron MR Controlled Evaluation (ADVANCED), CoreValve (Medtronic, Minneapolis, MN), Neurologic Complications of Unprotected Transcatheter Aortic Valve Implantation (Neuro-TAVI), Repositionable Percutaneous Replacement of Stenotic Aortic Valve Through Implantation of Lotus^TM^ Valve System (Boston Scientific, Marlborough, MA) - Randomized Clinical Evaluation (REPRISE II), and The Society of Thoracic Surgeons/American College of Cardiology Transcatheter Valve Therapy (STC/ACC TVT) registry studies further explored the risk of stroke in TAVR. The Effect of Bivalirudin on Aortic Valve Intervention Outcomes (BRAVO)-3 MRI, a prospective randomized evaluation of the TriGuard^TM^ HDH (Keystone Heart, Tampa, FL) Embolic Deflection Device During Transcatheter Aortic Valve Replacement (DEFLECT III), Claret Embolic Protection and Transcatheter Aortic Valve Implantation (CLEAN-TAVI), and Cerebral Protection in Transcatheter Aortic Valve Replacement (SENTINEL) trials investigated cerebral protection methods post-TAVR for stroke prevention.

In the PARTNER 1 trial, the stroke rate was greater in the TAVR group than in the SAVR group at one year (8.3% vs. 4.3%, P=0.04); the PARTNER 2 trial showed a lower risk of stroke at two years (6.2% and 6.4%, respectively). The NOTION and SURTAVI trials showed no significant difference in stroke rate between TAVR and SAVR at one year (13.1% vs 16.3%, respectively; p = 0.43). An increase in stroke rate after TAVR was found in the ADVANCED trial (5.6% at 31 days - two years) and CoreValve trial (4.3% in the late phase). The Neuro-TAVI trial showed ischemia after TAVR in 20% of patients at discharge. In the STC/ACC TVT Registry, the rate of post-procedure disabling stroke was 1.7% at 30 days. Finally, the BRAVO-3 MRI study showed no advantages between bivalirudin or unfractionated heparin in preventing cerebral lesion formation (65.5% vs 58.1%, respectively; p = 0.55). The DEFLECT III, CLEAN-TAVI, and SENTINEL trials assessed the safety and efficacy of transcatheter cerebral embolic protection devices (CEPDs); their results ranged from 50% improvement (CLEAN-TAVI) to none (DEFLECT III, SENTINEL), therefore, showing non-inferiority of cerebral embolic protection devices for TAVR patients.

The clinical trials studied in this paper showed a similar incidence of stroke in both groups, with the majority of TAVR patients developing cerebral lesions. With the addition of cerebral embolic protective devices, this incidence has decreased.

## Introduction and background

Aortic stenosis is a degenerative disease causing left ventricular outflow obstruction, decreased cardiac output, and subsequent mortality [1]. Once patients become symptomatic, 50% die within two years [2]. Aortic stenosis is increasing in prevalence, with 2.8% of adults over the age of 75 being affected [1]. Previously, the mainstay of treatment was surgical aortic valve replacement (SAVR). However, there are many contraindications to SAVR (most importantly, in patients who have an extreme surgical risk). In 2002, the first transcatheter aortic valve replacement (TAVR) was performed, allowing patients previously unable to undergo surgery to have an aortic valve replacement. Since then, many trials have sought to prove that TAVR was not inferior to SAVR. The Placement of Aortic Transcatheter Valves 1 (PARTNER 1) trial was the groundbreaking trial that showed lower mortality rates with TAVR in patients who could not undergo surgery or were at high risk for aortic valve replacement [2]. Since then, the Placement of Aortic Transcatheter Valves 2 (PARTNER 2) trial showed similar results in intermediate risk patients [3]. With the introduction of newer CoreValves™ (Medtronic, Minneapolis, MN) and the gaining expertise of physicians performing TAVR, TAVR has quickly surpassed SAVR with lower mortality rates and better outcomes than the traditional surgical approach [4]. However, research has shown conflicting reports about increased neurologic sequelae from TAVR [5].

Several trials have evaluated the benefits of TAVR vs. SAVR, the predictors of stroke after TAVR, and possible ways to reduce stroke numbers. This paper seeks to determine if there is a significant difference in post-procedural stroke rates between patients with aortic stenosis who undergo transcatheter aortic valve replacement versus surgical aortic valve replacement. If so, what can be done to alleviate this risk?

**Figure 1:**
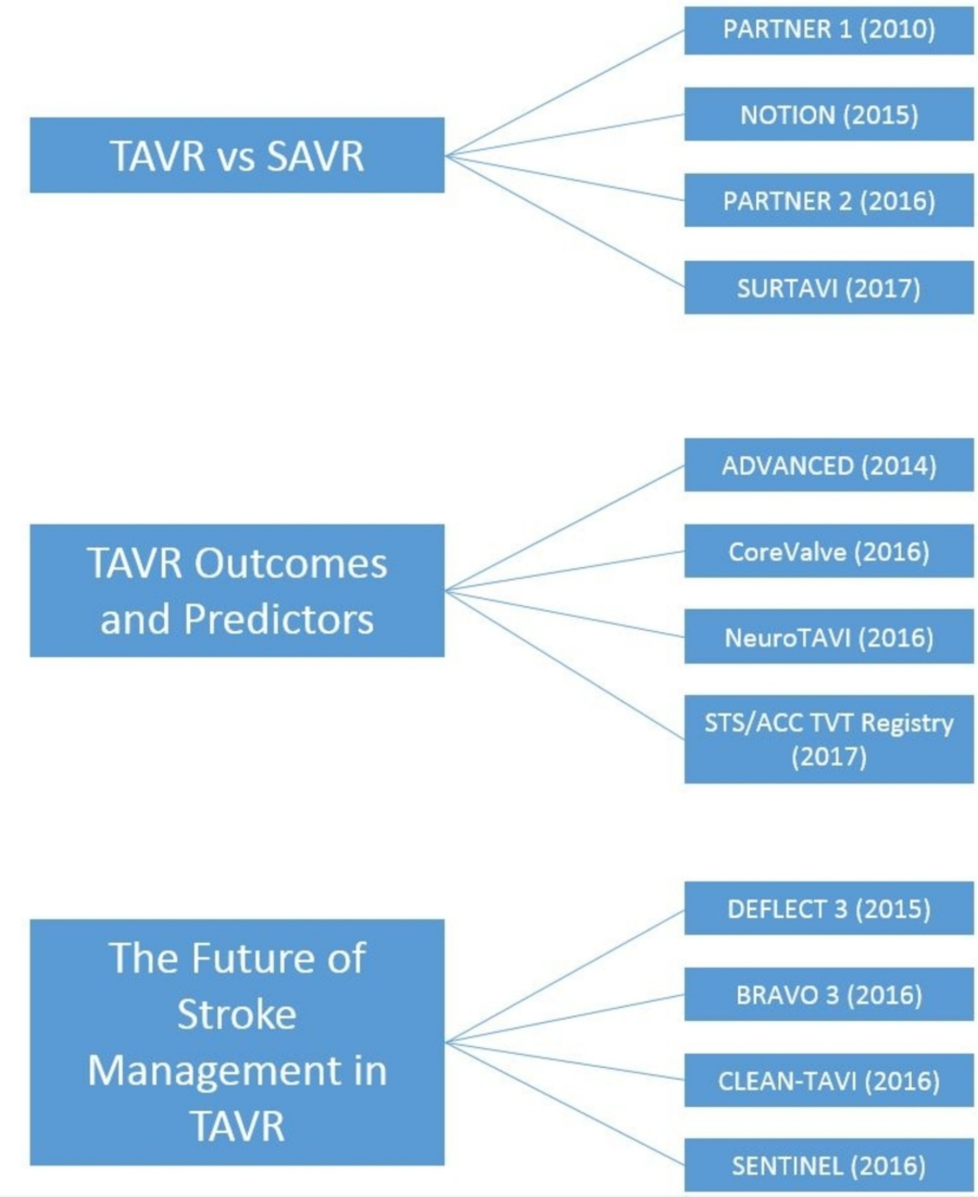
Schematic of Review Design TAVR: transcatheter aortic valve replacement; SAVR - surgical aortic valve replacement; PARTNER: Placement of Aortic Transcatheter Valves; NOTION: Nordic Aortic Valve Intervention; SURTAVI:  Surgical Replacement and Transcatheter Aortic Valve Implantation; ADVANCED: Action in Diabetes and Vascular Disease: Preterax and Diamicron MR Controlled Evaluation; NeuroTAVI: Neurologic Complications of Unprotected Transcatheter Aortic Valve Implantation; STS/ACC TVT: Society of Thoracic Surgeons/American College of Cardiology Transcatheter Valve Therapy; DEFLECT III: A prospective Randomized Evaluation of the TriGuard^TM^ HDH Embolic Deflection Device During Transcatheter Aortic Valve Replacement; BRAVO 3 MRI: Effect of Bivalirudin on Aortic Valve Intervention Outcomes; CLEAN-TAVI: Claret Embolic Protection and Transcatheter Aortic Valve Implantation; SENTINEL: Cerebral Protection in Transcatheter Aortic Valve Replacement

Our paper will summarize the four main trials which proved TAVR is non-inferior to SAVR: PARTNER 1, PARTNER 2, Nordic Aortic Valve Intervention (NOTION), and Surgical Replacement and Transcatheter Aortic Valve Implantation (SURTAVI) trials. It will also focus on trials exploring TAVR outcomes and predictors of stroke: Action in Diabetes and Vascular Disease: Preterax and Diamicron MR Controlled Evaluation (ADVANCED), CoreValve, Neurologic Complications of Unprotected Transcatheter Aortic Valve Implantation (Neuro-TAVI), Society of Thoracic Surgeons/American College of Cardiology Transcatheter Valve Therapy Registry (STS/ACC TVT Registry), and Repositionable Percutaneous Replacement of Stenotic Aortic Valve Through Implantation of Lotus™ Valve System – Randomized Clinical Evaluation (REPRISE II). Additionally, this paper looked at ways to alleviate stroke risk through anticoagulation and protection devices through the Effect of Bivalirudin on Aortic Valve Intervention Outcomes (BRAVO)-3 MRI, Claret Embolic Protection and Transcatheter Aortic Valve Implantation (CLEAN-TAVI), Cerebral Protection in Transcatheter Aortic Valve Replacement (SENTINEL), and Randomized Evaluation of the TriGuard™ (Keystone Heart, Tampa, FL) HDH Embolic Deflection Device During Transcatheter Aortic Valve Replacement (DEFLECT 3) trials (Figure 1). 

## Review

Comparing TAVR vs. SAVR

The PARTNER 1 trial was the first trial to objectively look at the differences in outcomes of patients with severe aortic stenosis receiving TAVR vs. traditional management (SAVR and medical management). Severe aortic stenosis was defined as an aortic valve area < 0.8 cm^2^ and a peak velocity > 4 meters per second (m/s) or a mean value gradient > 40 mmHg. The PARTNER 1 trial consisted of two separate trials using the SAPIEN heart system (Edwards Lifesciences Corp., Irvine, CA); the study endpoint for both trials was death from any cause at one year. Crossover of patient groups was not permitted [2].

The first arm of the trial assessed TAVR vs. SAVR outcomes in patients with severe aortic stenosis and high surgical risk. The high-risk TAVR vs. SAVR trial featured 699 patients, 492 in the transfemoral vs. aortic valve replacement (AVR) control, and 207 receiving either transapical TAVR or AVR control with 1:1 randomization. In the high-risk operable cohort, the one year rate of death was 24.2% in the TAVR group and 26.8% in the SAVR group (*p *= 0.44). Neurologic events (stroke and transient ischemic attack) were greater in the TAVR group at one year (8.3% vs. 4.3%, *p *= 0.04) [6]. The second arm of the trial assessed TAVR vs. standard therapy (medical management and/or balloon aortic valvuloplasty) in high-risk patients who could not undergo surgery and had a cohort of 358 patients and 1:1 randomization. In the high-risk non-operable group, the 30-day rate of mortality was 5% in the TAVR group and 2.8% in the standard therapy group and 30.7% in the TAVR group and 50.7% in the standard therapy group at one year. Neurologic events were also greater in the TAVR cohort at one year (7.8% vs 3.9%, *p *= 0.18) [2]. In conclusion, the PARTNER 1 trial showed that TAVR carried double the risk of neurologic sequelae but showed a decreased overall rate of mortality [6].

There were several weaknesses in the PARTNER 1 trial. First, the TAVR procedure was relatively new at the time; many of the physicians participated in the trial without extensive experience in TAVR placement. Second, there was a 5% dropout rate for the transcatheter patients. Third, there was not enough data to draw conclusions about differences in all outcomes (including stroke) regarding the transfemoral vs. transapical TAVR approach [6]. Lastly, the PARTNER 1 trial only looked at high-risk patients, potentially introducing selection bias and preventing its indications from being translated to low-risk and intermediate-risk patients [2, 6].

Low-risk and intermediate-risk patient outcomes were addressed in the NOTION trial, a multicenter, randomized, non-blinded trial which evaluated the safety and effectiveness of TAVR by using a self-expanding prosthesis compared with the traditional SAVR. The primary outcome of the study evaluated the composite of myocardial infarction (MI), stroke, or all-cause mortality between the two groups within the first year after the intervention. A total of 276 patients were included in the study; 142 received TAVR and 134 received SAVR. The mean age of the patients was 79.1 ± 4.8 years with 81.8% of the patients considered low-risk. The study was non-blinding, increasing the risk of analysis bias. However, the NOTION trial highlighted that there was no significant difference in the primary outcome between TAVR and SAVR groups within the first year after the intervention (13.1% vs. 16.3%, respectively; *p *= 0.43) [7].

The PARTNER 2 trial also served to compare intermediate risk patient outcomes in TAVR and SAVR. The trial enrolled 2,032 intermediate risk patients (as defined by a risk model developed by the Society of Thoracic Surgeons) and utilized the SAPIEN replacement valve. Imaging and clinical findings were used to place patients in either the transfemoral (76.3%) or the transthoracic TAVR approach (23.7%). Computer randomization entered patients into either TAVR or SAVR with 1:1 ratio. In contrast to the PARTNER 1 trial, the PARTNER 2 showed a lower risk of postoperative stroke complications from TAVR when compared to SAVR (6.2%, vs 6.4%, respectively) at two years. The rate of death from any cause was also lower in the TAVR group (16.7% vs 18%, respectively) at the two-year follow-up. The authors concluded that TAVR was non-inferior to SAVR in intermediate risk patients [3].

The PARTNER 2 trial had similar limitations to the PARTNER 1 trial in that there was a dropout rate; 94 patients did not undergo their scheduled procedure. Second, with continuous advancements of the SAPIEN valve system, the SAPIEN XT (Edwards Lifesciences Corp., Irvine, CA) valve used in this trial had since been replaced by the SAPIEN 3. Third, the long-term durability data of TAVR was still not addressed. The PARTNER 2 trial excluded all patients who had a stroke or transient ischemic attack (TIA) within six months prior to their procedure. It is unknown if this contributed to the PARTNER 2 trial showing a lower stroke risk in TAVR patients when compared to SAVR, contrary to the PARTNER 1 trial. Finally, the PARTNER 2 trial could only be applied to intermediate-risk patients [3].

The SURTAVI trial was similar to the PARTNER 2 trial, except it investigated the use of the Medtronic CoreValve or Evolut R™ (Medtronic, Minneapolis, MN) replacement valves instead of the SAPIEN system. The primary endpoint was all-cause mortality or disabling stroke within two years; secondary endpoints of interest included incidence of peri-procedural neurological injury in 30 days, the incidence of stroke and TIAs at two years, and the incidence of major adverse cardiovascular and cerebrovascular events at two years. In the SURTAVI trial, 1,660 patients were randomized to the two treatment groups, and there were no significant differences in mortality, including death from disabling stroke at 30 days (95% confidence interval (CI) (-2.8, 0.7)), at 12 months (95% CI (-3.5, 2.1)), and at 24 months (95% CI (-5.2, 2.3)). The only statistically significant difference in outcomes was in the incidence of stroke within 30 days in the TAVR vs SAVR group (95% CI (-4.2, -0.2)). The authors concluded that TAVR using the CoreValve system was non-inferior to SAVR in intermediate risk patients with aortic stenosis. Note that the study was not designed to determine if TAVR was superior to SAVR in this population group [8].

In response to the findings of the SURTAVI and PARTNER 2 trials, the American Heart Association/American College of Cardiology updated management guidelines in 2017 to give TAVR a Class IIa indication (reasonable alternative to surgical AVR) for severe aortic stenosis in intermediate surgical risk patients [8-9]. However, the correlation between TAVR and stroke outcomes deserves further exploration.

TAVR outcomes

The ADVANCED trial looked at 996 patients who underwent TAVR in order to further explore TAVR outcomes using the CoreValve [4]. The previous PARTNER 1 study used only a SAPIEN valve [2]. Pertinent to our paper, it looked at neurological events zero to one-day post-TAVR, two to 30 days post-TAVR, and 31-730 days post-TAVR. The trial found a 1.4% stroke rate at zero to one-day postop, 3.0% at two to 30 days postop, and 5.6% at 31 days - two years postop. The major stroke rate was 0.5%, 1.2%, and 2.9%, respectively. There were no variables that predicted zero to one-day postop stroke. However, female sex (*p *= 0.02), higher Society of Thoracic Surgery (STS) scores (*p *= 0.04), and major vascular complications (*p *= 0.01) were predictive in determining stroke risk two to 30 days postop. Additionally, prior coronary artery bypass grafting (CABG) was an indicator of stroke risk 31+ days postop (*p *= 0.003). The authors concluded that sex, STS scores, major vascular complications, and prior CABG were the only factors indicative of future stroke post-TAVR. However, it is important to note that the ADVANCED trial patients had lower overall STS scores than the PARTNER trial patients, that the overall rate of stroke post-TAVR was still relatively low (even with a cohort of 996 patients), and that unlike the PARTNER trial’s use of the SAPIEN valve system, the ADVANCED trial used a CoreValve System and used physicians with experience in TAVR. It is unknown if the physicians’ expertise with TAVR contributed to a lower overall stroke rate in the ADVANCED trial when compared to the PARTNER trial, or if the use of the CoreValve was a significant contributing factor. The ADVANCED trial can only be used to compare patients with a CoreValve and did not look at SAVR patients [5].

Patients enrolled in the CoreValve US Extreme Risk and High Pivotal Trials (both randomized control studies), as well as a Continued Access Study treated with self-expanding CoreValve bioprosthesis, were studied in the paper by Kleiman et al., with a focus placed on neurologic events following TAVR [10]. Analysis of stroke outcomes was categorized as early phase (zero to 10 days) and late phase (11 - 365 days). The incidence of stroke in the early phase was 4.1%; in the late phase, the incidence of stroke was 4.3%. Significant predictors of stroke in the early phase (days zero to 10) were the following: National Institutes of Health Stroke Scale (NIHSS) score > 0, history of cerebrovascular accident (CVA) or transient ischemic attack (TIA), history of peripheral vascular disease, no history of CABG, the presence of angina, body mass index < 21 kg/m^2^, and a fall within six months. Procedural predictors of stroke were the following: time in operating room, rapid pacing, and repositioning of the valve with a snare. The only significant predictor of stroke in the late phase (days 11 - 365) was a fall within the past six months. Echocardiography, computed tomography angiography (CTA) imaging, and clinical site experience were not significant predictors of stroke in the early or late phase [10]. Limitations included the absence of more extensive descriptions of the morphology of calcification in the aortic arch and ascending aorta, which could have been used to further explore possible predictors of stroke. However, that study suggested the frequency of stroke could be higher than those reported in previous registry studies [10].

The Neuro-TAVI Trial was a prospective cohort multicenter study that evaluated the frequency of cerebral ischemic lesions using diffusion-weighted magnetic resonance imaging (DW-MRI), neurologic injury, and cognitive dysfunction in subjects undergoing TAVR without cerebral protection in the US. The primary outcomes included the presence, number, and volume of new post-procedure DW-MRI lesions, as well as the degree of cognitive change (defined by mean change and any worsening in score from baseline to each follow-up). A total of 34 patients underwent post-procedure DW-MRI in which 94% of patients showed brain lesions with a mean of 10.4 ± 15.3 lesions per patient. In patients with evidence of cerebral infarction by DW-MRI, the worsening of either the NIHSS neurologic assessment or the Montreal Cognitive Assessment (MoCA) occurred in 59.4% of patients at pre-discharge and in 40.7% at 30 days. Using paper and pencil neuropsychological testing at baseline and at 30 days, there was a consistent decrease in cognitive measures in 20 - 56% of patients. Furthermore, there was a 6.8% (n = 3) periprocedural stroke rate (one disabling and two non-disabling strokes based on 30-day assessment). The major limitation of the study was the absence of pre-procedural DW-MRI for comparative analysis. In conclusion, the Neuro-TAVI trial highlighted that TAVR resulted in embolic insults to the brain in most patients with 20% of patients having new clinically evident neurologic impairment confirmed by DW-MRI at discharge and 15% of patients at 30 days. In addition, the trial showed that 33% of patients had a decrease in cognitive measures by the Montreal cognitive assessment (MoCA) score after TAVR, confirming the need to prevent or lower the risk of brain injury during the TAVR procedure [11].

The REPRISE II trial monitored one-year outcomes with the Lotus™ transcatheter aortic valve (Boston Scientific, Marlborough, MA) in 120 patients, which was designed with the previous REPRISE I design and placement limitations in mind. This prospective single-arm multicenter trial followed the outcomes of patients at seven days (or discharge), 30 days, three months, six months, and one year. Neurological status was also assessed pre- and post-procedure. Findings stratified by the New York Heart Association (NYHA) functional class showed an increased improvement in clinical outcome and mortality. However, the rate of disabling stroke at 30 days post-procedure was 1.7%; at one year, it was 3.4%. These values were comparable to that of the CoreValve and SAPIEN/SAPIEN XT trials, indicating that the stroke rate was not mitigated by the more sophisticated design of the valve. Limitations of the trial included that it was not randomized or double-blinded. Additionally, there was no control group of patients in this trial who did not receive a Lotus transcatheter aortic valve [12].

Due to the newness of TAVR, the STS/ACC TVT Registry was used to analyze poor outcomes of TAVR and their relationship with site-specific experience in performing TAVR. The registry included 42,988 commercial procedures from 395 hospitals. A volume-outcome model was designed to split the TAVR volume of each hospital into quartiles, and a case sequence approach was used to analyze the data. Individual sites that contributed to greater than one quartile served as their own controls [13]. There was a statistically significant decrease for most major adverse outcomes within the first 100 procedures completed at a site; after this first 100, there was a general decline in adverse outcomes. Stroke incidence, in particular, showed a decrease from 2.03% to 1.66%, but this decrease was not statistically significant (p = 0.14). Additionally, confidence limits for stroke were broad due to the low rate of reports citing strokes in the registry. One limitation of this study was its use of only one TAVR model. The analysis was also limited to commercially-approved technology, so hospitals participating in trials had experience with the valves prior to the recording of the procedures in the registry [13].

Prevention of stroke

The formation of thrombotic materials leading to cerebral embolization is common in TAVR due to the manipulation of stenotic aortic valves during the procedure. Stroke preventative methods, such as anticoagulants and hardware, are commonly used in other cardiovascular procedures. As a result, it is relevant to investigate the applicability of similar preventative methods during TAVR to lower the incidence of cerebral emboli.

The BRAVO-3 MRI study is a part of the BRAVO-3 trial, a double-blind randomized, controlled trial comparing bivalirudin with unfractionated heparin (UFH) in patients undergoing transfemoral TAVR to evaluate whether parenteral procedural anticoagulation affects cerebral embolization. The primary outcome evaluated new cerebral emboli on DW-MRI. A total of 60 patients with severe aortic stenosis and high surgical risk were randomized into bivalirudin (n = 29) and UFH (n = 31) groups. In total, 61.7% of the patients showed one or more new cerebral emboli on DW-MRI and did not differ between the bivalirudin and UFH groups (65.5% vs 58.1%, respectively; *p *= 0.55). A weakness of the BRAVO-3 MRI study was a lack of DW-MRI at baseline and at 30 days for comparative analysis. In conclusion, the BRAVO-3 MRI study highlighted that the majority of the patients developed one or more new cerebral emboli after TAVR regardless of the procedural anticoagulation therapy to prevent cerebral embolization [14].

A developing area of research around TAVR involves the use of transcatheter cerebral embolic protection devices (CEPD) during the procedure in order to prevent cerebral embolic material from passing through the cerebral arteries. These devices are inserted at the beginning of the surgery and positioned so that the filter covers the three outlets of the cerebral arteries in the aortic arch. Blood flow is maintained, but the particulate matter is collected in the filter. The device is removed after the TAVR procedure is completed.

A number of recent studies have evaluated the safety and efficacy of these CEPD devices in TAVR patient populations. The DEFLECT III trial utilized the TriGuard HDH (Keystone Heart, Tampa, FL) embolic deflection device in a multi-center, single-blinded randomized control trial. This exploratory study evaluated high and extreme risk patients undergoing TAVR procedures. In a sample size of 85 patients, 39 were randomized to TAVR without CEPD and 46 to TAVR with CEPD. The primary endpoint was major adverse cardiovascular and cerebrovascular events (MACCE), and secondary endpoints included neurological changes and frequency, number, per-patient average, and volume of lesions on DW-MRI. Although the only statistically significant result was a 44% decrease in single lesion volume with the TriGuard CEPD, all average endpoints were improved in the protected group compared to the unprotected group. The authors stated that these results, although insignificant, would be used to inform the design of a randomized trial using the TriGuard device [15].

Similarly, the CLEAN-TAVI trial assessed the effect of the Claret Montage™ Dual Filter System (Claret Medical, Santa Rosa, CA) on the number and volume of cerebral lesions in TAVR patients. The study was a single-center, blinded, randomized control trial that compared a group with CEPD to a group given routine post-TAVR management without CEPD. A total of 50 patients were randomized to the CEPD arm while another 50 were assigned to the control. Patients were high-risk surgical candidates selected by the institution’s heart team. The trial found statistically significant reductions in the lesion numbers in the CEPD group compared to the control at day two (eight vs 16, respectively; *p *= 0.0023) and at day seven (five vs 10, respectively; *p *= 0.012). In addition, the total lesion volume was also significantly decreased in the CEPD group at both endpoints. The authors concluded that the use of a CEPD reduced the frequency and volume of ischemic cerebral lesions but that further studies were needed to assess the effect on neurological and cognitive function [16]. The results of the CLEAN-TAVI trial have limited applicability to the general population due to the small sample size, subjective patient selection, and extensive exclusion criteria. Future studies could investigate the CEPD in wider patient populations with larger sample sizes; in addition, formal neurological workup could be performed to assess for neurological outcomes [16].

The SENTINEL trial evaluated the Claret Medical Sentinel® device (Claret Medical, Santa Rosa, CA) through a double-blinded randomized control trial of TAVR patients who were high-risk surgery candidates at 17 centers. This study assigned 363 patients in a 1:1:1 ratio to either the device, the device with imaging, or a control with imaging to assess for major cardiac and cerebrovascular events at 30 days and for a reduction in new lesion volume on MRI scans at two and seven days from baseline. Because the rate of major adverse cardiac and cerebrovascular events was not statistically significant between the control and device groups, the authors concluded that the study met the criteria for non-inferiority. The lesion volume decreased from 178 mm^3^ in controls to 102.8 mm^3^ in the device arms (*p *= 0.33). Although this was not a statistically significant reduction, this trial concluded that the Sentinel device was safe to use. These results may have been affected by the variability in committee-driven patient selection at 17 different centers and use of four different valve replacement systems; future studies should control for these variables [17].

## Conclusions

This review first discussed the results of the PARTNER 1, NOTION, PARTNER 2, and SURTAVI trials to compare the risk of stroke in patients with aortic stenosis undergoing SAVR or TAVR. The results of these studies led to the 2017 AHA/ACC guidelines allowing for the use of TAVR in both high-risk and intermediate-risk surgical candidates for aortic valve replacement. However, rates of stroke post-TAVR had yet to be elucidated. The Neuro-TAVI study further explored outcomes in TAVR patients and found that 94% had brain lesions on DW-MRI and 59.4% had diminished neurocognitive abilities post-procedurally.The risk of stroke specifically was followed through the results of the ADVANCED, CoreValve, REPRISE II, and STS/ACC TVT Registry studies. The REPRISE II study looked for relationships between valve design and outcome. The STS/ACC TVT Registry explored the influence of site experience on TAVR outcomes; the results showed a statistically significant decrease in all outcomes, except stroke with an increased site experience. In response to the risk of postoperative stroke, this review also evaluated stroke prevention strategies in future TAVR patients, both medical and procedural. Anticoagulation therapy is a mainstay of transcatheter procedural guidelines, and the BRAVO-3 trial showed no significant advantages in choosing between bivalirudin or unfractionated heparin. However, the rapid expansion of anticoagulant therapies on the market indicates future studies could be performed on the safety and efficacy outcomes of these therapies. The development of a number of cerebral protection devices was discussed as an area of future research for procedural stroke prevention. The DEFLECT III, CLEAN-TAVI, and SENTINEL trials assessed the safety and efficacy of transcatheter cerebral embolic protection devices. Although results ranged from 50% improvement to no significant improvement in lesion number or volume, the authors established non-inferiority of CEPDs for TAVR patients and determined areas of interest for future research. As this technology further develops, it may prove to be a promising stroke preventative measure during TAVR procedures.
